# Alexithymia and emotional distress in patients with asthma: A case-control Study

**DOI:** 10.1192/j.eurpsy.2025.1246

**Published:** 2025-08-26

**Authors:** I. Baati, N. Regaieg, F. Cherif, D. Mnif, K. Amor, I. Feki, F. Guermazi, J. Masmoudi

**Affiliations:** 1 Psychiatry A Department, University of Sfax, Faculty of medicine of Sfax, Sfax, Tunisia

## Abstract

**Introduction:**

Alexithymia is characterized by difficulty in verbally expressing emotions, a trait commonly observed in patients with psychosomatic symptoms, such as those with asthma. These individuals struggle to identify their emotions and are more prone to developing somatic health issues.

**Objectives:**

- To study the prevalence of alexithymia, anxiety and depression in asthma patients
- To identify the relationship between these aspects.

**Methods:**

It was a cross-sectional, case-control, descriptive and analytical study, conducted in the pulmonology department at Hedi Chaker University Hospital in Sfax. The study involved 50 asthma patients and a control group consisting of 50 healthy nurses. Data collection was based on a sociodemographic and clinical questionnaire, as well as two psychometric scales: the Toronto Alexithymia Scale 20 (TAS-20) and the Hospital Anxiety and Depression Scale (HADS).

**Results:**

The mean age of the patients was 44.08 ± 12.78 years and the male-to-female ratio (M/F) was 0.92.

More than half of the patients (62%) had experienced an acute severe asthma requiring hospitalization. The disease was controlled in 70% of the cases.

The prevalence of alexithymia was 40% in the asthma patients and 36% in the control group, with no significant difference between the two groups (p = 0.68).

The prevalence of anxiety was 38% in patients and 34% in the control group with a significant difference between the two groups (p = 0.012).

The prevalence of depression was 38% in patients and 28% in the control group with a significant difference between the two groups (p = 0.002).

Alexithymic patients were significantly more anxious (p = 0.01) and more depressed (p = 0.01) than non-alexithymic patients.

**Conclusions:**

Patients with ashtma present high rates of anxiety, depression and alexithymia. This latter does not appear to be a determining factor in asthma. However, it seems to be associated with the emotional distress caused by anxiety and depression. Timely screening for emotional distress and its early management is essential in this population.

**Disclosure of Interest:**

None Declared

